# Next-generation sequencing for sensitive detection of *BCR-ABL1* mutations relevant to tyrosine kinase inhibitor choice in imatinib-resistant patients

**DOI:** 10.18632/oncotarget.8010

**Published:** 2016-03-09

**Authors:** Simona Soverini, Caterina De Benedittis, Katerina Machova Polakova, Jana Linhartova, Fausto Castagnetti, Gabriele Gugliotta, Cristina Papayannidis, Manuela Mancini, Hana Klamova, Marzia Salvucci, Monica Crugnola, Alessandra Iurlo, Francesco Albano, Domenico Russo, Gianantonio Rosti, Michele Cavo, Michele Baccarani, Giovanni Martinelli

**Affiliations:** ^1^ Institute of Hematology “L. e A. Seràgnoli”, Department of Experimental, Diagnostic and Specialty Medicine, University of Bologna, Bologna, Italy; ^2^ Institute of Hematology and Blood Transfusion, Prague, Czech Republic; ^3^ Oncology-Hematology Department, “S. Maria delle Croci” Hospital, Ravenna, Italy; ^4^ Hematology, Parma University Hospital, Parma, Italy; ^5^ Division of Haematology, Fondazione IRCCS Ca’ Granda Ospedale Maggiore Policlinico, Milano, Italy; ^6^ Hematology Section, Department of Emergency and Organ Transplantation, University of Bari, Bari, Italy; ^7^ Unit of Blood Disease and Stem Cell Transplantation, Department of Clinical and Experimental Sciences, University of Brescia, Brescia, Italy

**Keywords:** chronic myeloid leukemia, acute lymphoblastic leukemia, BCR-ABL1, tyrosine kinase inhibitors, Next-generation sequencing

## Abstract

In chronic myeloid leukemia (CML) and Philadelphia-positive (Ph+) acute lymphoblastic leukemia (ALL) patients who fail imatinib treatment, *BCR-ABL1* mutation profiling by Sanger sequencing (SS) is recommended before changing therapy since detection of specific mutations influences second-generation tyrosine kinase inhibitor (2GTKI) choice. We aimed to assess i) in how many patients who relapse on second-line 2GTKI therapy next generation sequencing (NGS) may track resistant mutations back to the sample collected at the time of imatinib resistance, before 2GTKI start (switchover sample) and ii) whether low level mutations identified by NGS always undergo clonal expansion. To this purpose, we used NGS to retrospectively analyze 60 imatinib-resistant patients (CML, *n* = 45; Ph+ ALL, *n* = 15) who had failed second-line 2GTKI therapy and had acquired *BCR-ABL1* mutations (Group 1) and 25 imatinib-resistant patients (CML, *n* = 21; Ph+ ALL, *n* = 4) who had responded to second-line 2GTKI therapy, for comparison (Group 2). NGS uncovered that in 26 (43%) patients in Group 1, the 2GTKI-resistant mutations that triggered relapse were already detectable at low levels in the switchover sample (median mutation burden, 5%; range 1.1%–18.4%). Importantly, none of the low level mutations detected by NGS in switchover samples failed to expand whenever the patient received the 2GTKI to whom they were insensitive. In contrast, no low level mutation that was resistant to the 2GTKI the patients subsequently received was detected in the switchover samples from Group 2. NGS at the time of imatinib failure reliably identifies clinically relevant mutations, thus enabling a more effective therapeutic tailoring.

## INTRODUCTION

At the time of resistance to therapy with the tyrosine kinase inhibitor (TKI) imatinib, a proportion of Philadelphia chromosome-positive (Ph+) leukemia patients ranging from 30 to 70% – depending on disease phase and type – are found to carry mutations in the *BCR-ABL1* kinase domain (KD) when analyzed by Sanger sequencing (SS) [1, 2]. Almost a decade of clinical experience with the second-generation TKIs (2GTKIs) dasatinib and nilotinib has confirmed initial, *in vitro* predictions and has consolidated the knowledge that some imatinib-resistant mutations retain insensitivity to one or both – hence are relevant for 2GTKI choice [3–7]. Patients positive for F317L/V/I/C, T315A or V299L should receive nilotinib rather than dasatinib [8]; patients positive for Y253H, E255K/V, F359V/I/C should receive dasatinib rather than nilotinib [8]; patients positive for the T315I mutation may benefit from the recently approved third-generation TKI ponatinib [9].

Thus, albeit different TKIs have different indications, availability, cost and tolerability profiles to be taken into account [10], *BCR-ABL1* mutation status is an important component of the therapeutic decision algorithm for imatinib-resistant patients [8, 10–14]. Conventional Sanger sequencing (SS) is the gold standard and the currently recommended method for diagnostic *BCR-ABL1* KD mutation screening [8, 15]. However, SS has limited sensitivity and cannot robustly identify mutated populations < 20%.

We have recently set up an assay for targeted Next-Generation Sequencing (NGS) of the *BCR-ABL1* KD and have validated its accuracy, precision, and linearity for detection of any sequence variation down to 1% [16]. In a small series of chronic myeloid leukemia (CML) and Ph+ acute lymphoblastic leukemia (ALL) patients with multi-TKI-resistant disease, NGS showed that *BCR-ABL1* KD mutation status may be much more complex than SS shows, with one or multiple low level mutants often detectable in addition to dominant mutants [16]. We then reasoned that, from a clinical standpoint, the setting in which the greater sensitivity of NGS could be most useful is when a Ph+ leukemia patient with a non-optimal response has to be shifted to second-line therapy. We thus undertook a study aimed to explore i) in how many patients who relapse on second-line 2GTKI therapy NGS would be able to track resistant mutations back to the switchover sample; ii) whether low level mutations identified by NGS undergo clonal expansion if the 2GTKI to whom they are insensitive happens to be chosen – and can thus be regarded as a reliable predictor of subsequent failure. To this purpose, we used NGS to retrospectively analyze 60 patients who had failed second-line therapy and had acquired *BCR-ABL1* KD mutations as assessed by SS (Group 1) and 25 patients who had responded to second-line therapy, for comparison (Group 2).

## RESULTS

### Mutations detected by SS at switchover and at the time of subsequent relapse on dasatinib or nilotinib (Group 1)

**Figure 1:**
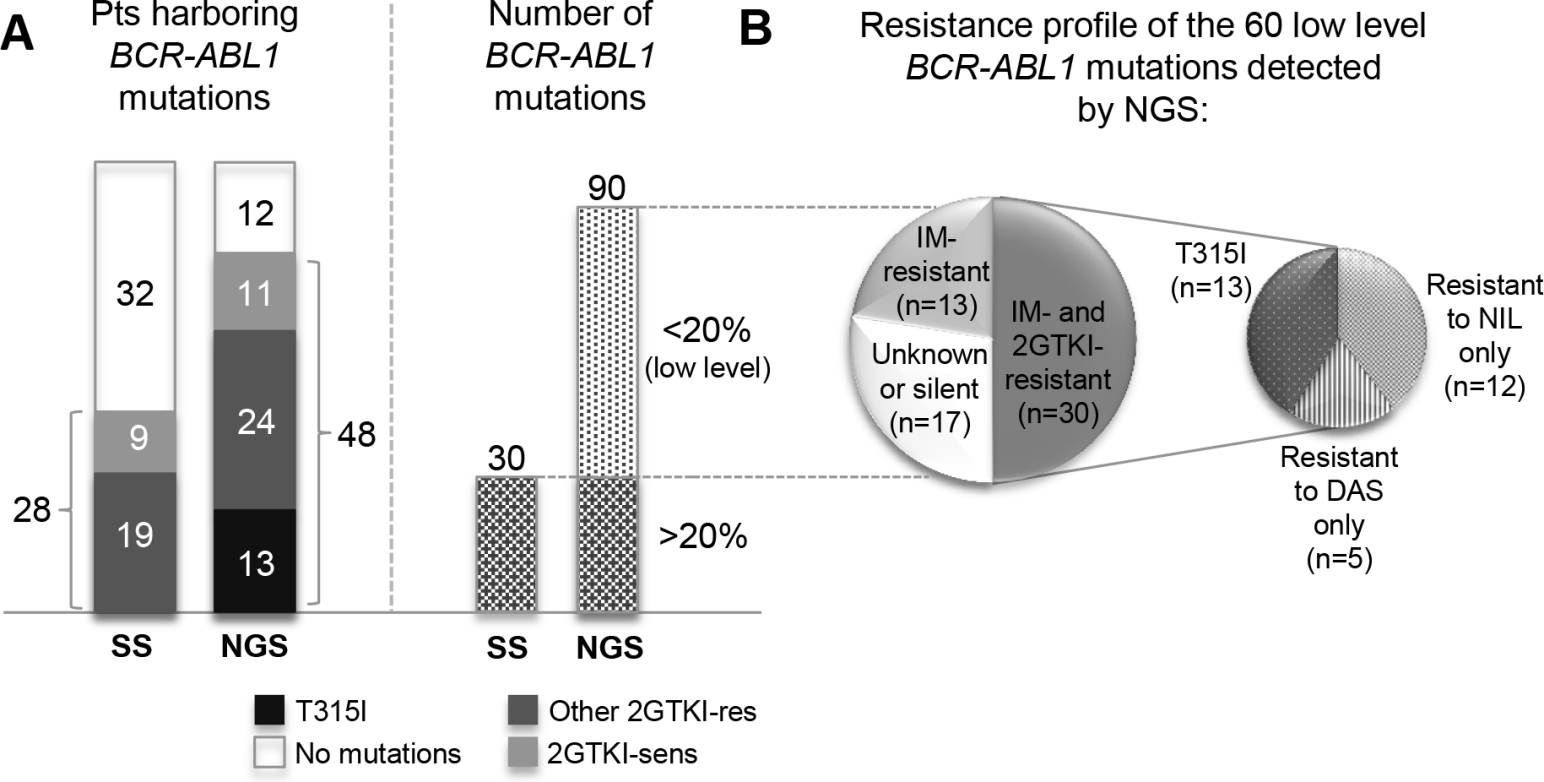
(**A**) Comparison between the number of patients found to harbor BCR-ABL KD mutations by NGS as against SS. At the time of imatinib failure, patients were switched to dasatinib or nilotinib after routine *BCR-ABL1* KD mutation screening, so no patient positive for the T315I by SS is included in this study. (**B**) Breakdown of the 60 low level mutations detected by NGS according to their resistance profile. Number of mutations within each category is in brackets. ‘Mutations resistant to nilotinib only’ include Y253H, E255K, E255V, F359V/C. ‘Mutations resistant to dasatinib only’ refer to F317L (since no low level V299L was detected at switchover). Abbreviations: res, resistant; sens, sensitive; IM, imatinib; DAS, dasatinib, NIL, nilotinib; 2GTKI, second generation tyrosine kinase inhibitor.

Switch to second-line dasatinib (*n* = 39) or nilotinib (*n* = 21) had been prompted by imatinib failure (defined according to European LeukemiaNet [ELN] recommendations [17, 18]) in all the 60 patients included in Group 1 (Supplementary Table S1). Choice of dasatinib over nilotinib or *vice versa* had been performed after assessment of *BCR-ABL1* KD mutation status by SS, so no patient positive for the T315I mutation by SS at the time of imatinib resistance is included in this series. At the time of switchover, overall, SS had identified mutations (*n* = 30) in 28/60 (47%) patients (Figure 1A and Supplementary Table S2). Among them, 19 patients had dasatinib- or nilotinib-resistant mutations that guided TKI selection. The remaining 9 patients with mutations resistant to imatinib only as well as the 32 patients with no evidence of mutations by SS were switched to dasatinib or nilotinib based on drug availability, comorbidities, or at the physician's discretion.

Dasatinib-resistant mutations found by SS to be acquired at the time of second relapse included T315I in 21 patients, F317L or –V in 14 patients, both T315I and F317L in 3 patients, V299L in 1 patient. Nilotinib-resistant mutations included Y253H in 6 patients, E255K or –V in 5 patients, F359V or -I in 3 patients; 1 additional patient was found to have acquired both a T315I and an E255K and 1 patient was found to have simultaneously acquired a T315I, an E255K and an F359I (Supplementary Table S2). Mutations were detected by SS after a median of 6 months (range, 1–48) from switchover.

### Mutations detected by NGS at switchover and at the time of subsequent relapse on dasatinib or nilotinib (Group 1)

**Figure 2:**
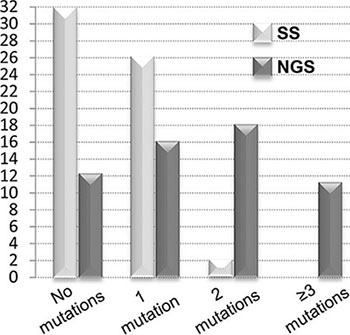
Number of patients harboring no mutations, 1 mutation, 2 mutations and 3 or more mutations by NGS as against SS NGS provided a more accurate picture of *BCR-ABL1* KD mutation status.

**Figure 3:**
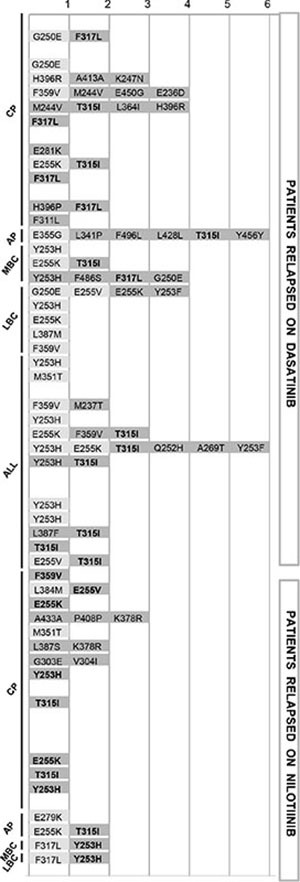
Mutations detected by NGS as against SS detailed for each of the 60 patients (Y axis), grouped by disease phase/type and by 2GTKI received Light grey indicates mutations detectable both by SS and by NGS, dark grey indicates mutations detectable by NGS only. The bold font highlights the low level mutations that became detectable by SS at the time of relapse, two to nine months later. In all cases in which the 2GTKI to whom they were insensitive happened to be selected, low level mutations invariably expanded, alone or in combination with pre-existing mutations (See also Table S2). Abbreviations: CP, chronic phase; AP, accelerated phase; MBC, myeloid blast crisis; LBC, lymphoid blast crisis; ALL, acute lymphoblastic leukemia. Definitions of AP and BC as per ELN criteria.

In 26/60 (43%) patients, NGS uncovered that the dasatinib- or nilotinib-resistant mutations that later triggered relapse were already detectable, at low levels (> 1% but < 20%) in the switchover samples. NGS reanalysis indeed painted a more accurate picture of *BCR-ABL1* KD mutation status (Figures 1–3 and Supplementary Table S2). Twenty out of 32 patients found to have no mutations by SS turned out to carry one or more mutations by NGS. In 15 of these patients, low level mutations missed by SS (median mutation burden, 5.8%; range: 1.4%–18.4%) could have been relevant for TKI choice. Also, 15 of the 28 patients with mutations detectable by SS had additional low level mutations detectable by NGS. In 11 of these patients, low level mutations (median mutation burden, 3.75%; range: 1.11%–16.9%) could have been relevant for TKI choice.

Overall, 48/60 (80%) patients were found to harbor mutations at any level by NGS. NGS detected all the 30 mutations that had been previously identified by SS, plus 60 low level mutations. Of the 60 low level mutations found by NGS only, 17 were silent or had an unknown resistance profile, 13 were known to be resistant to imatinib only, and 30 were known to confer resistance also to dasatinib or nilotinib (T315I, *n* = 13; other nilotinib-resistant [F359V, E255K, E255V, Y253H], *n* = 12; other dasatinib-resistant [F317L], *n* = 5) (Figure 1B).

The knowledge of the mutations emerged at relapse revealed that none of the low level dasatinib- or nilotinib-resistant mutations detected by NGS at switchover failed to expand whenever the patient received the 2GTKI to whom they were insensitive (Figure 3; Supplementary Table S2).

At relapse, NGS detected low level mutations additional to the dominant one(s) in 19/60 patients. NGS confirmed the 86 mutations already found by SS and uncovered 28 additional low level mutations (Supplementary Table S2).

### Mutations detected by NGS at switchover in patients who did not experience subsequent relapse (Group 2)

We then wondered what we would find if we analyzed by NGS switchover samples of imatinib-resistant patients who did respond to subsequent second-line treatment. To explore whether the 1% threshold we chose to adopt might lead to the identification of transient mutants that are not going to undermine TKI effectiveness, switchover samples of 25 randomly selected cases who achieved a stable response to second-line treatment with dasatinib or nilotinib were also analyzed, for comparison. By NGS, a total of 20 mutations were detected in 16 patients – including 7 mutations that had already been identified in 7 patients by SS, plus 13 low level mutations in 10 patients (Supplementary Table S3). No low level mutation resistant to the 2GTKI that each patient actually received was identified. The 2 patients in whom NGS identified a low level F317L mutation received nilotinib. The patient in whom NGS identified a low level Y253H was switched to dasatinib.

## DISCUSSION

Many TKI options are currently available for the treatment of imatinib-resistant patients. Although safety, tolerability and price are important components of the decision algorithm, 2GTKIs have some Achilles heels in terms of resistant mutations that, when detected, shift the balance towards one rather than another [8]. Mutation profiling of imatinib-resistant patients is routinely performed with SS [8, 15]. In the past years, other strategies had been explored in an attempt to improve upon SS in terms of sensitivity of mutation detection. PCR-based approaches like amplification refractory mutational system (ARMS)- or Ligation (L)-PCR [19–24] achieve the greatest sensitivity (up to one mutated transcript in ten thousand total *BCR-ABL1* transcripts) and rely just on a real-time PCR instrument, but they require expensive panels of fluorescent primers/probes and many reactions must be run in parallel in order to cover all the nucleotide substitutions that may lead to dasatinib- and nilotinib-resistance. More importantly, when used in longitudinal studies to investigate the dynamics of low level mutations, ARMS- or L-PCR results failed to prove clinically useful as reliable indicators of subsequent treatment failure [21, 22, 25, 26]. For this reason, the ELN expert panel agreed to not recommend these methodologies for routine diagnostic use [8]. More recently, a strategy of primer extension coupled with mass spectrometry-based detection (Sequenom MassARRAY) [27] was used to retrospectively investigate the presence of a panel of 31 clinically relevant *BCR-ABL1* mutations in baseline samples of imatinib-resistant CML patients who were enrolled in the phase 2 clinical studies of dasatinib and nilotinib [28]. Low level mutations (lower detection limit of the MassARRAY was reported to be, on average, 0.2%) could be detected in half of the patients and were indeed predictive of subsequent outcome. In patients who fail TKI therapy, the presence of mutations below the lower detection limit of SS is not unexpected. Depending on the type of resistance (molecular vs. cytogenetic or hematologic) an outgrowing mutant population may not have yet achieved an abundance of 15–20%. Also the timing of sampling is an important variable – if sampling has occurred after TKI withdrawal and/or during or after a cycle of cytoreductive therapy, a previously dominant mutant population may have temporarily receded because of the lack of selective pressure [20, 29]. In both scenarios, mutations would thus be missed by SS. The Sequenom MassARRAY, however, is an expensive research instrumentation conceived for large-scale genotyping studies of dozens to hundreds of loci in hundreds to thousands of samples and it is therefore unsuitable for routine, prospective use [27]. We reasoned that, currently, NGS represents the most suitable alternative. NGS is a robust, powerful and versatile technology that is becoming accessible to a wider and wider number of diagnostic laboratories [30–34]. We had already set up an NGS-based assay for *BCR-ABL1* KD mutation screening and demonstrated the accuracy, precision, and linearity for detection of any sequence variation down to 1% [16]. We thus decided to inventory, among the Ph+ leukemia patients referred to our laboratories for routine BCR-ABL KD analysis, all those who had acquired mutations eliciting resistance to second-line dasatinib or nilotinib therapy. We aimed to reanalyze these patients by NGS in order to assess in how many cases these mutations (and 2GTKI-resistant mutations in general) could be tracked back to the switchover samples. Our results demonstrate that in a not negligible proportion of cases, an NGS-based mutation screening could have changed therapeutic decisions towards a TKI more likely to be successful. This is particularly relevant now that the recent approval of ponatinib offers significant chances to rescue patients with the T315I or with multiple mutations predicted to result in insensitivity to both dasatinib and nilotinib. In 15 out of 32 patients in which SS had failed to detect any mutation, NGS identified 2GTKI-resistant mutations that would have guided TKI selection. Among the remaining 28 patients with mutations detectable by SS, 11 had additional low level mutations important for a more appropriate selection of the second-line treatment strategy. This is also not unexpected, since distinct resistant subclones following independent ‘escape routes’ have been described to arise in some patients. The relative proportion between these different subclones is, again, a time-dependent, dynamic variable influenced by clonal competition and other factors. Let us consider, for example, patient no. 22 (see Figure 3 and Supplementary Table S2), carrying an E255K at 23% and a T315I at 16%. The E255K mutation alone (as per SS results) would suggest choosing dasatinib, but the finding of an additional T315I mutation (detectable by NGS only) shifts the balance towards ponatinib as the only reasonable TKI option. This strengthens the importance of performing a comprehensive and sensitive screening for *BCR-ABL1* KD mutations in all patients at switchover, since this will offer the greater chance to choose the most effective TKI available.

Most importantly, all the low level dasatinib- or nilotinib-resistant mutations detected by NGS at switchover (down to 1% abundance) expanded whenever the patient happened to receive the 2GTKI to whom they were insensitive. Additionally, when we analyzed by NGS the switchover samples of patients who did respond to second-line treatment with dasatinib or nilotinib, no low level mutation that was resistant to the 2GTKI the patient received was detected. This suggests that mutants with 1–20% abundance, detectable by NGS only, are clinically relevant – a *sine-qua-non* condition for NGS to be used to inform therapeutic decisions. This is in line with many recent studies in other forms of leukemia or in solid tumors, showing that very minor subclones detectable by NGS do harbour the same predictive and prognostic value as the major ones [35–37].

Taken together, our results demonstrate that NGS is a convincing alternative to SS for routine *BCR-ABL1* KD mutation screening of Ph+ leukemia patients. Sample centralization in reference laboratories (as is already the case in many countries) would reduce costs and turnaround times, aligning them to those of SS-based analysis. Our data provide a strong *rationale* for clinical studies aimed to prospectively optimize the integration of NGS in the clinical management of Ph+ leukemia patients and lay the foundations for the revision of the ELN recommendations for *BCR-ABL1* KD mutations analysis in CML.

## METHODS

### Patients and samples

Between 2006 and 2013, as the Italian and Czech central reference laboratories for *BCR-ABL1* mutation analysis, we followed a total of 79 imatinib-resistant patients with CML or Ph+ ALL who failed second-line dasatinib or nilotinib therapy and had evidence of newly acquired mutations at relapse as assessed by SS. In 60 cases, leftover RNA was available for NGS reanalysis, that was performed on matched samples collected at the of time of switchover and at the time of subsequent relapse, for a total of 120 samples. The main characteristics of these 60 patients (CML, *n* = 45; Ph+ ALL, *n* = 15) (Group 1) are presented in Supplementary Table S1. Definition of AP and BC was according to the ELN criteria [10]. Because of the design of the study, we included in this analysis some of the patients already reported in [16].

To explore whether the greater sensitivity of NGS might lead to the identification of dasatinib- or nilotinib-resistant mutations that will not outgrow and cause relapse, switchover samples of 25 randomly selected cases who achieved a stable response to second-line treatment with dasatinib (*n* = 15) or nilotinib (*n* = 10) (Group 2) were also analyzed, for comparison.

All the patients provided written informed consent, in accordance with the Declaration of Helsinki. Institutional Review Boards approval was obtained.

### Conventional sequencing of the *BCR-ABL1* KD

SS of the *BCR-ABL1* KD was performed on an ABI PRISM 3730 (Applied Biosystems, Foster City, CA) as previously reported. [38, 39].

### NGS of the *BCR-ABL1* KD

RNA was converted to cDNA with the Transcriptor High-Fidelity cDNA Synthesis kit (Roche Applied Science, Mannheim, Germany). To select for the translocated *ABL1* allele, a first step of amplification was performed by polymerase chain reaction (PCR) with a forward primer either on *BCR* exon 1a (in case of e1a2 *BCR-ABL1* fusion) or on *BCR* exons 12–13 border (in case of b2a2 or b3a2 *BCR-ABL1* fusions) and a reverse primer on *ABL1*, exon 10. A second amplification step was then performed to generate four partly overlapping amplicons covering the kinase domain of *ABL1* – tagged with a 10-base ‘barcode’ sequence (multiplex identifier) for sample pooling. Primer sequences and PCR protocols have been previously reported [16]. Amplifications were done using the FastStart High-Fidelity PCR System kit (Roche Applied Science). The amplicons were purified with Agencourt AMPure XP beads (Beckman Coulter, Krefeld, Germany) and quantified using the Quant-iT PicoGreen dsDNA kit (Invitrogen, Carlsbad, CA, USA) according to the Amplicon Library Preparation Method Manual (454 Life Sciences, Branford, CT). Equimolar pooling of amplicons was followed by clonal amplification of the single DNA molecules on beads (emulsion PCR) according to the emPCR Amplification Method Manual, Lib-A (454 Life Sciences). After emulsion breaking and bead recovery, enriched DNA-containing beads were sequenced on a GS Junior instrument according to the Sequencing Method Manual for the Titanium sequencing kit (454 Life Sciences). NGS was performed on a Roche GS Junior (454 Life Sciences) according to the manufacturer's instructions. Amplicon Variant Analyzer ver2.7 (454 Life Sciences) and Sequence Pilot ver4.0.1 (JSI-Medical Systems, Kippenheim, Germany) were used to align reads to the reference *ABL1* sequence (GenBank accession no. X16416.1) and to calculate variant frequencies. The presence of all relevant mutations was also manually verified by inspection of individual flowgrams at the corresponding positions, with particular attention to homopolymeric regions. NGS runs were designed for high sensitivity mutation calling: the target sequence coverage was at least 5000 clonal reads for each nucleotide position of interest. Practically, it ranged from 4850 to 9543 across the samples analyzed. This allowed to achieve a detection limit as low as 1% of *BCR-ABL1* transcripts and to test whether this could reliably predict for emerging, clinically actionable mutations. The estimated cut-off for significant mutation calling was set to 1.0% based on error distribution analysis (negative binomial distribution analysis of errors was applied to NGS data of cDNA samples of non-mutated controls, as detailed in [40]). Sensitivity, accuracy and reproducibility of our NGS-based *BCR-ABL1* mutation screening assay had already been demonstrated, as reported in [16]. Robustness, reproducibility and clinical utility of the 454 technology for NGS of candidate leukemia-associated genes have also extensively been addressed by Grossmann et al. [32] and in the framework of the IRON (Interlaboratory RObustness of Next-generation sequencing)-I and -II international studies [30, 33].

### Definitions

‘Switchover sample’ defines the sample collected at the time of imatinib resistance, immediately before second-line 2GTKI therapy start. Mutations detectable by NGS only (generally, mutations with an abundance of 20% or lower) have been defined ‘low level mutations’ throughout the manuscript. Y253H, E255K, E255V, V299L, F317L, F359V or T315I, conferring resistance to dasatinib, nilotinib or both, have been referred to as ‘mutations relevant for TKI choice’.

## SUPPLEMENTARY FIGURES AND TABLES


