# Supplementary material for: Next-generation sequencing for sensitive detection of BCR-ABL1 mutations relevant to tyrosine kinase inhibitor choice in imatinib-resistant patients
Source: Oncotarget. 2016 Mar 9;7(16):21982–90. doi: 10.18632/oncotarget.8010 (PMC5008338; doi:10.18632/oncotarget.8010)
Supplement: Supplementary file 1 [file oncotarget-07-21982-s001.pdf]

Next-generation sequencing for sensitive detection of *BCR-ABL1* mutations relevant to tyrosine kinase inhibitor choice in imatinib-resistant patients

Supplementary Materials

Supplementary Table S1: Main features of group 1 patients

Pts, total	60
Median age, years (range)	54 (25–78)
Male to female ratio	35:25
Disease phase/type:	
CML	45
chronic phase	32
accelerated phase	3
myeloid blast crisis	4
lymphoid blast crisis	6
Ph+ ALL	15
Pts who failed 2nd-line dasatinib	39
Pts who failed 2nd-line nilotinib	21
Median time from diagnosis to 2nd-line TKI therapy start, months (range)	18 (3–72)
Median time from 2nd-line TKI therapy start to relapse, months (range)	6 (1–48)

Abbreviations: Pts, patients; CML, chronic myeloid leukemia; Ph+ ALL, Philadelphia chromosome-positive acute lymphoblastic leukemia.

Supplementary Table S2: SS and NGS results at the time of switchover and at the time of subsequent resistance to second-line dasatinib or nilotinib

N	Disease Phase	Sex	Age	TKI	Mut by SS	Mut by NGS	Response at the time of analysis
1	CP-CML	F	27	IM	No mutations	No mutations	No CHR after 3 months on IM
				DAS	T315I (~100%)	T315I (94.8%)	Progression to LBC after 2 months on DAS
2	CP-CML	M	69	IM	No mutations	**F359V (16.5%)**	Loss of MMR after 39 months on IM
				NIL	F359V (~60%)	F359V (59.3%)	Loss of complete CyR after 3 months on NIL
3	CP-CML	M	62	IM	G250E (~100%)	G250E (93.7%), **F317L (1.8%)**	Minor CyR after 12 months on IM
				DAS	G250E (~50%), F317L (~40%)	G250E (45.5%), F317L (37.5%), H295H (4.9%), C330C (1.5%)	No CyR after 13 months on DAS
4	CP-CML	F	60	IM	L384M (~100%)	L384M (87.0%), **E255V (15.1%)**	Loss of complete CyR after 24 months on IM
				NIL	E255V (~100%)	E255V (80.7%), L384M (14.4%)	No CHR after 3 months on NIL
5	CP-CML	F	60	IM	No mutations	No mutations	Minor CyR after 12 months on IM
				DAS	F317L (~100%)	F317L (100.0%)	Loss of CHR after 12 months onDAS
6	CP-CML	M	49	IM	No mutations	**E255K (18.4%)**	Loss of complete CyR after 48 months on IM
				NIL	E255K (~100%)	E255K (79.9%)	Minimal CyR after 6 months on NIL
7	CP-CML	F	57	IM	G250E (~100%)	G250E (99.5%)	Loss of complete CyR after 36 months on IM
				DAS	F317L (~100%)	F317L (99.5%)	No CyR after 3 months on DAS
8	CP-CML	F	71	IM	H396R (~100%)	H396R (99.6%), A413A (1.7%), K247N (1.1%)	Partial CyR after 18 months on IM
				DAS	F317L (~20%)	F317L (20.9%), H396R (3.5%)	Minimal CyR after 6 months on DAS
9	CP-CML	M	44	IM	No mutations	A433A (1.5%), P408P (1.4%), K378R (1.3%)	No CyR after 12 months on IM

				NIL	T315I (~70%)	T315I (65.9%), Y253H (16.7%), W235R (3.8%), F497L (3.1%), T406I (2.4%), Q477Q (2.4%), F486S (2.3%), D363N (1.5%)	No CyR after 13 months on NIL
10	CP-CML	M	66	IM	M351T (~100%)	M351T (99.8%)	No CyR after 12 months on IM
				NIL	Y253H(~100%)	Y253H (95.6%), M351T (3.2%)	No CyR after 22 months on NIL
11	CP-CML	F	60	IM	F359V (~80%)	F359V (87.0%), M244V (5.6%), E450G (2.6%), E236D (1.1%)	Loss of MMR after 20 months on IM
				DAS	F317L (~100%)	F317L (100%), F486L (6.8%)	Loss of MMR after 43 months on DAS
12	CP-CML	F	75	IM	No mutations	M224V (3.2%), **T315I (3.1%),** L364I (2.0%), H396R (1.4%)	No complete CyR after 69 months on IM
				DAS	M244V (~100%), T315I (~20%)	M244V (100%), T315I (18.0%)	No CyR after 25 months on DAS
13	CP-CML	M	53	IM	No mutations	L387S (1.6%), K378R (1.0%)	Loss of complete CyR after 20 months on IM
				NIL	T315I (~20%)	T315I (21.1%), Y253H (3.6%)	Loss of complete CyR after 27 months on NIL
14	CP-CML	F	55	IM	No mutations	G303E (1.2%), V304I (1.1%)	No complete CyR after 50 months on IM
				NIL	E255K (~100%)	E255K (89.0%)	No CyR after 6 months on NIL
15	CP-CML	M	43	IM	No mutations	**F317L (11.3%)**	Loss of MMR after 39 months on IM
				DAS	F317L (~50%)	F317L (50.0%), L451P (1.8%), K294E (1.6%)	No CyR after 1 month on DAS
16	CP-CML	M	34	IM	No mutations	No mutations	Loss of complete CyR after 22 months on IM
				DAS	V299L (~100%)	V299L (99.0%), T394A (2.0%)	Loss of MMR after 24 months on DAS
17	CP-CML	M	40	IM	No mutations	E281K (1.3%)	No complete CyR after 18 months on IM
				DAS	T315I (~20%)	T315I (22.0%)	Loss of complete CyR after 12 months on DAS
18	CP-CML	M	64	IM	No mutations	**Y253H (5.1%)**	No CyR after 6 months on IM
				NIL	Y253H (~100%)	Y253H (99.5%)	No CyR after 6 months on NIL

19	CP-CML	M	51	IM	No mutations	No mutations	Loss of MMR after 48 months on IM
				NIL	F359I (~100%)	F359I (99.2%)	Loss of complete CyR after 18 months on NIL
20	CP-CML	M	44	IM	No mutations	**T315I (8.3%)**	Loss of complete CyR after 18 months on IM
				NIL	T315I (~100%)	T315I (99.7%)	Loss of hematologic response after 3 months on NIL
21	CP-CML	M	31	IM	No mutations	No mutations	Loss of MMR after 24 months on IM
				NIL	E255V (~100%)	E255V (99.7%)	Loss of complete CyR after 12 months on NIL
22	CP-CML	F	37	IM	E255K (~25%)	E255K (23.2%), **T315I (16.9%)**	Loss of CHR after 9 months on IM
				DAS	E255K (~100%), T315I (~100%)	E255K (99.6%), T315I (98.6%)	No CHR after 1 month on DAS
23	CP-CML	F	70	IM	No mutations	**F317L (6.5%)**	Loss of complete CyR after 24 months on IM
				DAS	F317L (~100%)	F317L (99.5%)	Progression to AP after 6 months on DAS
24	CP-CML	M	50	IM	No mutations	No mutations	Loss of complete CyR after 36 months on IM
				NIL	T315I (~100%), E255K (~50%), F359I (~30%)	T315I (92.0%), E255K (48.2%), F359I (32.5%)	Progression to MBC after 24 months on NIL
25	CP-CML	F	49	IM	No mutations	No mutations	No complete CyR after 18 months on IM
				NIL	F359V (~100%)	F359V (99.7%)	Loss of CHR after 9 months
26	CP-CML	M	41	IM	No mutations	No mutations	No CyR after 3 months on IM
				DAS	F317L (~100%)	F317L (ttc > tta) (80.7%), F317L (ttc > ctc) (9.73%)	Loss of CHR after 6 months on DAS
27	CP-CML	M	65	IM	No mutations	**E255K (15.4%)**	Loss of complete CyR after 72 months on IM
				NIL	E255K (~100%)	E255K (97.8%)	Loss of CHR after 3 months on NIL
28	CP-CML	M	57	IM	No mutations	**T315I (5.0%)**	Loss of CHR after 24 months on IM
				NIL	T315I (~100%)	T315I (100.0%)	Progression to MBC after 3 months on NIL
29	CP-CML	F	67	IM	No mutations	H396P (14.1%), **F317L (ttc > tta) (1.4%)**	Loss of complete CyR after 24 months on IM

				DAS	F317L (~100%)	F317L (ttc > ctc) (55.2%), F317L (ttc > tta) (44.7%)	Loss of CHR after 3 months on NIL
30	CP-CML	F	59	IM	No mutations	**Y253H (4.4%)**	Loss of MMR after 60 months on IM
				NIL	Y253H (~100%)	Y253H (99.8%)	Loss of CCyR after 3 months on NIL
31	CP-CML	F	62	IM	No mutations	No mutations	No complete CyR after 24 months on IM
				NIL	Y253H (~50%)	Y253H (45.5%)	Loss of complete CyR after 48 months on NIL
32	CP-CML	F	52	IM	No mutations	F311L (5.2%)	No MMR after 24 months on IM
				DAS	T315I (~70%)	T315I (66.5%)	Loss of complete CyR after 36 months on DAS
33	AP-CML	M	49	IM	E355G (~60%)	E355G (43.8%), L341P (19.8%), F496L (19.6%), L428L (11.0%), **T315I (10.2%),** Y456Y (4.8%)	Progression to AP after 9 months on IM
				DAS	T315I (~50%)	T315I (42.6%), F317L (1.3%)	Progression to MBC after 1 month on DAS
34	AP-CML	M	44	IM	E279K (~50%)	E279K (55.4%)	Progression to AP after 6 months on IM
				NIL	E279K (~50%), T315I (~50%)	E279K (48.8%), T315I (49.8%)	Loss of CHR after 6 months on NIL
35	AP-CML	M	39	IM	E255K (~100%)	E255K (98.0%), **T315I (1.7%)**	Progression to AP after 18 months on IM
				NIL	E255K (~100%), T315I (~100%)	E255K (99.0%), T315I (99.0%)	No HR after 1 month on NIL
36	MBC-CML	M	32	IM	Y253H (~100%)	Y253H (100.00%)	Progression to MBC after 22 months on IM
				DAS	F317L (~60%), T315I (~40%)	F317L (55.3%), T315I (35.7%), T315A (7.0%), Y253H (4.1%)	No CHR after 3 months on DAS
37	MBC-CML	M	48	IM	E255K (~100%)	E255K (97.5%), **T315I (3.7%)**	No CHR after 2 months on IM
				DAS	E255K (~100%), T315I (~100%)	E255K (97.7%), T315I (96.8%)	No CHR after 1 month on DAS

38	MBC-CML	F	59	IM	No mutations	Y253H (17.8%), F486S (11.6%), **F317L (9.6%),** G250E (8.2%)	Progression to MBC after 36 months on IM
				DAS	F317L (~100%)	F317L (60.9%)	No CHR after 1 month of DAS
39	MBC-CML	M	43	IM	F317L (~100%)	F317L (99.9%), **Y253H (17.4%)**	Progression to MBC after 44 months on IM
				NIL	F317L (~100%), Y253H (~100%)	F317L (99.9%), Y253H (99.9%)	No CHR after 2 months on NIL
40	LBC-CML	M	56	IM	G250E (~40%), E255V (~20%)	G250E (34.2%), E255V (21.1%), E255K (2.3%), Y253F (1.1%)	Progression to LBC after 32 months on IM
				DAS	T315I (~30%)	T315I (28.9%)	No CHR after 2 months on DAS
41	LBC-CML	F	63	IM	Y253H (~100%)	Y253H (99.9%)	Progression to LBC after 48 months on IM
				DAS	Y253H (~50), F317L (~50)	Y253H (54.9%), F317L (54.4%)	Loss of CHR after 6 months on DAS
42	LBC-CML	F	26	IM	E255K (~100%)	E255K (98.8%)	Progression to LBC after 9 months on IM
				DAS	E255K (~100%), T315I (~100%)	E255K (99.8%), T315I (99.4%), L273S (1.4%)	No CHR after 1 month on DAS
43	LBC-CML	M	78	IM	L387M (~30%)	L387M (34.1%)	Loss of CHR after 6 months on IM
				DAS	L387M (~100%), T315I (~50%), M318V (~50%), F317L (~50%), Y320N (~50%)	L387M (96.3%), T315I (51.5%), M318V (51.2%), F317L (45.2%), Y320N (44.8%)	Loss of complete CyR and CHR after 14 months on DAS
44	LBC-CML	M	28	IM	F359V (~60%)	F359V (57.9%)	Progression to LBC after 3 months on IM
				DAS	F359V (~100%), F317I (~100%)	F317I (92.4%), F359V (90.1%), F317L (4.3%)	Loss of CHR after 9 months on DAS
45	LBC-CML	M	55	IM	F317L (~100%)	F317L (99.5%), **Y253H (1.11%)**	Progression to LBC after 32 months on IM
				NIL	F317L (~100%), Y253H (~100%)	Y253H (98.9%), F317L (98.1%)	No CHR after 1 month on NIL

46	ALL	M	26	IM	Y253H (~100%)	Y253H (99.8%)	Hematologic relapse after 6 months on IM
				DAS	Y253H (~100%), T315I (~50%)	Y253H (99.7%), T315I (40.3%), N336S (5.4%), W405R (1.9%)	Hematologic relapse after 9 months on DAS
47	ALL	M	64	IM	M351T (~100%)	M351T (99.9%)	Hematologic relapse after 12 months on IM
				DAS	M351T (~100%), F317L (~50%)	M351T (99.8%), F317L (53.3%)	Hematologic relapse after 12 months on DAS
48	ALL	M	37	IM	No mutations	No mutations	Hematologic relapse after 15 months on IM
				DAS	F317L (~100%)	F317L (100.0%)	Hematologic relapse after 6 months on DAS
49	ALL	M	36	IM	F359V (~100%)	F359V (99.8%), M237T (2.1%)	Hematologic relapse after 26 months on IM
				DAS	F359V (~100%), T315I (~100%)	F359V (99.3%), T315I (98.8%), Y449Y (10.5%)	Progressive disease after 1 month on DAS
50	ALL	F	50	IM	Y253H (~30%)	Y253H (28.4%)	Molecular relapse after 12 months on IM
				DAS	Y253H (~100%), T315I (~100%)	Y253H (87.7%), T315I (86.2%)	Hematologic relapse after 2 months on DAS
51	ALL	F	33	IM	E255K (~60%)	E255K (54.8%), F359V (13.8%), **T315I (3.8%)**	Hematologic relapse after 6 months on IM
				DAS	T315I (~100%)	T315I (99.4%)	Progressive disease after 5 months on DAS
52	ALL	M	25	IM	Y253H (~50%), E255K (~20%)	Y253H (56.0%), E255K (19.9%), **T315I (8.3%)**, Q252H (6.3%), A269T (3.6%), Y253F (1.4%)	Hematologic relapse after 9 months on IM
				DAS	T315I (~100%)	T315I (91.0%)	Progressive disease after 3 months on DAS
53	ALL	M	74	IM	No mutations	Y253H (2.5%), **T315I (2.5%)**	Hematologic relapse after 6 months on IM

				DAS	Y253H (~50%), T315I (~50%), F317L(ttc > tta) (~20%)	Y253H (49.0%), T315I (49.0%), F317L(ttc > tta) (20.0%), F317L(ttc > ctc) (5.0%)	Progressive disease after 2 months on DAS
54	ALL	F	78	IM	No mutations	No mutations	Hematologic relapse after 12 months on IM
				DAS	T315I (~100%)	T315I (99.5%)	Hematologic relapse after 7 months on DAS
55	ALL	F	60	IM	No mutations	No mutations	Hematologic relapse after 5 months on IM
				DAS	T315I (~100%)	T315I (92.5%)	Hematologic relapse after 3 months on DAS
56	ALL	F	64	IM	Y253H (~100%)	Y253H (100.00%)	Molecular relapse after 15 months on IM
				DAS	T315I (~100%)	T315I (100.00%), V289A (3.0%)	Hematologic relapse after 12 months on DAS
57	ALL	F	68	IM	Y253H (~100%)	Y253H (98.1%)	Hematologic relapse after 6 months on IM
				DAS	T315I (~100%), Y253H (~60%)	T315I (99.6%), Y253H (63.3%)	Hematologic relapse after 3 months on DAS
58	ALL	M	59	IM	No mutations	L387F (1.4%), **T315I (1.4%)**	Hematologic relapse after 9 months on IM
				DAS	L387F (~100.0%), T315I (~100.0%)	L387F (100.0%), T315I (91.2%)	Hematologic relapse after 2 months on DAS
59	ALL	M	44	IM	No mutations	**T315I (2.4%)**	Hematologic relapse after 15 months on IM
				DAS	T315I (~80%)	T315I (70.7%)	Hematologic relapse after 3 months on DAS
60	ALL	F	66	IM	E255V (~100%)	E255V (92.8%), **T315I (2.1%)**	Hematologic relapse after 18 months on IM
				DAS	E255V (~50%), T315I (~30%)	E255V (50.4%), T315I (24.3%)	Progressive disease after 1 month on DAS

Mutation burden is indicated in brackets; estimation was based on relative mutated to wild-type peak height in the sequencing output chromatogram or on the ratio between the number of reads harbouring the substitution and the total reads covering the corresponding nucleotide position for SS and NGS, respectively. When the same F317L mutation was found to result from different nucleotide sustitutions in distinct clones, codon change is specified in brackets. Mutations that triggered dasatinib or nilotinib failure and were already detectable by NGS at switchover are in bold. Abbreviations: CP, chronic phase; MBC, myeloid blast crisis; LBC, lymphoid blast crisis; CyR, cytogenetic response; MMR, major molecular response; CHR, complete hematologic response; IM, imatinib; DAS, dasatinib; NIL, nilotinib.

Supplementary Table S3: NGS results in the 25 imatinib-resistant patients who had an optimal response to second-line dasatinib/nilotinib therapy, analyzed for comparison

N	Disease Phase	Sex	Age	Mutation status by SS	Mutation status by UDS	Response at switchover	2nd-line TKI
1	CP-CML	M	39	No mutations	Y320H (1.5%), H295R (1.5%)	Loss of complete CyR after 36 months on IM	NIL
2	CP-CML	M	47	No mutations	No mutations	Loss of MMR after 48 months on IM	NIL
3	CP-CML	F	72	No mutations	No mutations	No complete CyR after 24 months on IM	DAS
4	CP-CML	M	66	H396P (~70%)	H396P (65.2%)	Loss of complete CyR after 44 months on IM	NIL
5	CP-CML	F	55	No mutations	L298R (1.4%)	No MMR after 24 months on IM	NIL
6	CP-CML	F	51	No mutations	No mutations	Loss of MMR after 38 months on IM	DAS
7	CP-CML	F	31	No mutations	S438F (2.8%)	Loss of MMR after 48 months on IM	DAS
8	CP-CML	M	69	No mutations	No mutations	No MMR after 18 months on IM	DAS
9	CP-CML	M	45	No mutations	No mutations	No MMR after 24 months on IM	NIL
10	CP-CML	M	52	No mutations	Y253C (2.0%), I432V (2.0%)	No complete CyR after 18 months on IM	DAS
11	CP-CML	F	73	No mutations	F317L (3.6%)	Loss of complete CyR after 60 months on IM	NIL
12	CP-CML	M	37	Y253H (~100%)	Y253H (98.5%)	Loss of complete CyR after 37 months on IM	DAS
13	CP-CML	F	44	No mutations	H396P (14.7%), F317L (1.4%)	Loss of complete CyR after 36 months on IM	NIL
14	CP-CML	M	65	No mutations	No mutations	No MMR after 26 months on IM	NIL
15	CP-CML	F	61	No mutations	No mutations	No CyR after 6 months on IM	NIL
16	CP-CML	M	53	No mutations	No mutations	Minor CyR after 12 months on IM	NIL
17	CP-CML	M	49	No mutations	F497L (18.1%)	No complete CyR after 24 months on IM	DAS
18	MBC-CML	M	64	L387M (~30%)	L387M (32.2%)	Progression to MBC after 89 months on IM	DAS
19	MBC-CML	F	70	Y253H (~30%)	Y253H (27.6%), P465L (1.6%)	Progression to MBC after 22 months on IM	DAS
20	MBC-CML	M	52	No mutations	No mutations	Progression to MBC after 89 months on IM	DAS
21	LBC-CML	M	53	Y253F (~50%)	Y253F (45.6%)	Progression to LBC after 17 months on IM	DAS
22	ALL	F	71	No mutations	P439S (7.8%), A474A (6.2%)	Molecular relapse after 14 months on IM	DAS

23	ALL	M	69	M351T (~100%)	M351T (100.0%)	Molecular relapse after 18 months on IM	DAS
24	ALL	M	66	No mutations	Y253H (2.1%)	Molecular relapse after 21 months on IM	DAS
25	ALL	M	59	M244V (~40%)	M244V (37.1%)	Loss of hematologic response after 12 months on IM	DAS

Definition of optimal response as per 2013 ELN recommendations. The main features (phase/type of disease, male to female ratio, median age, type of resistance to first-line imatinib therapy, type of 2GTKI administered, median time from diagnosis) do not differ significantly from the 60 patients who were resistant to second-line dasatinib or nilotinib. Median follow-up on second-line therapy, 16 months (range, 9–36). Abbreviations: CP, chronic phase; MBC, myeloid blast crisis; LBC, lymphoid blast crisis; CyR, cytogenetic response; MMR, major molecular response; IM, imatinib; DAS, dasatinib; NIL, nilotinib.
